# Management of Borderline Twin-to-Twin Transfusion Syndrome With Fetoscopic Laser Therapy in a Monochorionic Diamniotic Twin Pregnancy

**DOI:** 10.7759/cureus.101333

**Published:** 2026-01-12

**Authors:** Lorena Zijaj, Enkeleda Prifti, Anila Rexha Shahini, Arjan Kashami, Petra Kohlberger

**Affiliations:** 1 Obstetrics and Gynecology, Health Care Center, Tiranë, ALB; 2 Gynecology, Gynecological Obstetric University Hospital "Koço Gliozheni", Tiranë, ALB; 3 Cardiology, Health Care Center, Tiranë, ALB; 4 Obstetrics, Gynecological Obstetric University Hospital "Koço Gliozheni", Tiranë, ALB; 5 Gynecology, Medical University of Vienna, Vienna, AUT

**Keywords:** doppler ultrasound monitoring, fetoscopic laser therapy, monochorionic diamniotic twins, twin anemia-polycythemia sequence, twin-to-twin transfusion syndrome

## Abstract

Monochorionic diamniotic (MCDA) twin pregnancies are associated with a high risk of complications due to shared placental vascular anastomoses, which predispose fetuses to twin-to-twin transfusion syndrome (TTTS) and twin anemia-polycythemia sequence (TAPS). Early recognition of subtle hemodynamic alterations is essential, as borderline forms of these conditions may progress rapidly and jeopardize fetal well-being. This case report presents a 30-year-old gravida 2, para 1, with an MCDA twin pregnancy complicated by borderline TTTS and TAPS, who underwent fetoscopic laser therapy despite not meeting strict Quintero criteria. Ultrasound assessment at 22 weeks’ gestation revealed significant polyhydramnios and early Doppler abnormalities in the recipient twin, accompanied by mildly altered Doppler indices and borderline amniotic fluid reduction in the donor twin. Although diagnostic thresholds for classic Stage I TTTS or definitive TAPS were not fully met, the combination of symptomatic polyhydramnios, Doppler disturbances, and the patient’s high-risk maternal background, including chronic hypertension and a previous pregnancy complicated by severe preeclampsia and intrauterine growth restriction, prompted referral to a specialized fetal therapy center. Fetoscopic laser coagulation and amnioreduction were performed, resulting in rapid normalization of Doppler parameters and amniotic fluid volumes. Weekly post-procedural surveillance demonstrated stable fetal growth and restored hemodynamic balance. Cesarean delivery at 36 weeks’ gestation resulted in two healthy male neonates with favorable Apgar scores and an uncomplicated postpartum course. This case underscores the importance of individualized management and timely intervention in MCDA twin gestations, even when classical staging systems are not fully satisfied. Early detection of evolving hemodynamic compromise, strict adherence to standardized surveillance protocols, and multidisciplinary collaboration are crucial for optimizing maternal and perinatal outcomes in borderline TTTS and TAPS.

## Introduction

Monochorionic diamniotic (MCDA) twin pregnancies account for approximately 70-75% of monochorionic gestations and carry a significantly increased risk of perinatal morbidity and mortality due to placental vascular anastomoses that permit intertwin blood exchange [1]. These vascular connections may lead to severe complications such as twin-to-twin transfusion syndrome (TTTS) and twin anemia-polycythemia sequence (TAPS), which can rapidly compromise fetal hemodynamics if not diagnosed and managed promptly [1,2].

TTTS is a serious complication that affects 10-15% of monochorionic multiple pregnancies, which usually occurs between the 16th and 26th weeks of gestation. When untreated, the perinatal mortality is as high as 90% [3-5].

TTTS is traditionally diagnosed using the Quintero staging system, which is based on amniotic fluid discordance and the presence or absence of specific Doppler abnormalities. In contrast, TAPS is characterized by large intertwin differences in hemoglobin levels caused by minuscule arterio-venous anastomoses and is typically identified through discordant middle cerebral artery peak systolic velocities (MCA-PSV), often in the absence of amniotic fluid imbalance [6]. Although clear diagnostic criteria exist for both TTTS and TAPS, borderline or evolving forms pose substantial diagnostic and therapeutic challenges. In such cases, clinical judgment becomes essential, particularly when subtle ultrasound changes suggest impending deterioration despite criteria not being fully met.

Fetoscopic laser photocoagulation remains the gold standard treatment for moderate to severe TTTS and has been increasingly utilized in selected cases of TAPS or mixed presentations, especially when disease progression is anticipated [7]. Early referral to a specialized fetal therapy center is paramount, as timely intervention may significantly improve perinatal outcomes and reduce long-term neurodevelopmental sequelae.

The present case describes a 30-year-old patient with an MCDA twin pregnancy complicated by borderline TTTS and borderline TAPS, who underwent fetoscopic laser therapy despite not fully meeting the classical Quintero criteria. This report highlights the importance of early diagnosis, timely referral to a specialized fetal therapy center, and individualized management, which collectively contributed to a successful maternal and neonatal outcome.

## Case presentation

This case report presents the clinical course and management of an MCDA twin pregnancy complicated by borderline TTTS and TAPS. Although the case did not entirely fulfill the classical criteria for fetoscopic laser therapy, the procedure was performed with excellent post-laser evolution and favorable perinatal outcomes for both fetuses.

The patient was a 30-year-old gravida 2, para 1, with a history of one previous cesarean section performed at 29 weeks’ gestation for severe preeclampsia and intrauterine growth restriction (IUGR, 1020 g). In the current pregnancy, she was diagnosed with an MCDA twin gestation and was known to have chronic hypertension under pharmacological control.

**Figure 1:**
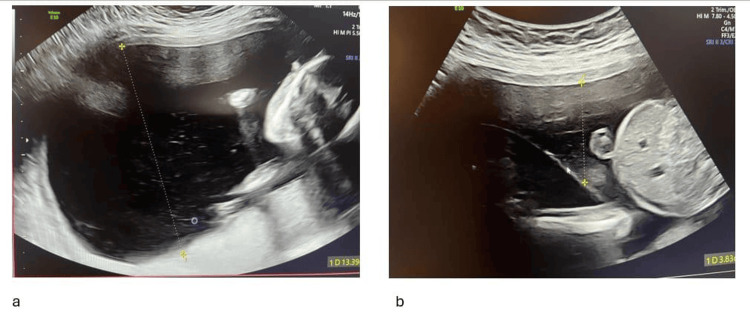
Recipient twin with polyhydramnios (DVP, 13 cm) (a) and donor twin with amniotic fluid volume at the lower limit of normal (DVP, 4 cm) (b). DVP: deepest vertical pocket

**Figure 2:**
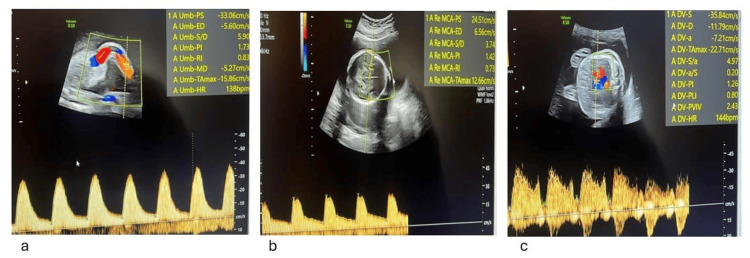
Doppler assessment of the donor twin showing normal umbilical artery flow (a), decreased middle cerebral artery peak systolic velocity with a cerebroplacental ratio <1.0 (b), and elevated ductus venosus resistance index with a preserved positive a-wave (c).

At 22 weeks of gestation, she was evaluated at the Department of Fetal-Maternal Medicine, University Hospital of Vienna, where a detailed ultrasonographic assessment revealed a viable cephalic fetus 1 with biometric measurements within normal range and a posterior placenta. The amniotic fluid was increased, with a deepest vertical pocket (DVP) of 13 cm, and the fetal bladder appeared distended (Figure 1). Doppler examination showed elevated resistance in the ductus venosus (pulsatility index for veins, PIV = 1.430) with a preserved a-wave, normal umbilical artery (UA) Doppler, and decreased MCA-PSV of 18.0 cm/s and a cerebroplacental ratio (CPR) of 0.661 (<1.0) (Figure 2).

**Figure 3:**
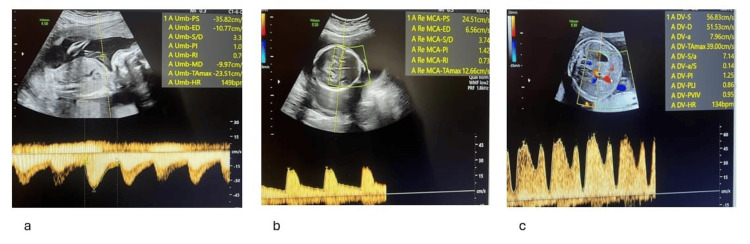
Doppler assessment of the recipient twin demonstrating normal umbilical artery flow (a), increased middle cerebral artery peak systolic velocity of approximately 1.5 multiples of the median (b), and elevated ductus venosus resistance index with a preserved positive a-wave (c).

Fetus 2 was viable in breech presentation with normal biometry and a posterior placenta. The amniotic fluid was at the lower limit of normal (DVP 4 cm), the bladder appeared normal, UA Doppler was normal, and the ductus venosus showed mildly increased resistance (PIV 1.360) with positive a-wave. The MCA-PSV measured approximately 1.5 multiples of the median (MoM) (Figure 3). The overall findings were consistent with borderline TTTS associated with borderline TAPS. As fetoscopic laser coagulation was not available at the Fetal-Maternal Medicine Department of the University Hospital of Vienna, the patient was referred to the Department of Obstetrics, University Hospital of Graz, for specialized management. At this center, she underwent fetoscopic laser therapy, amnioreduction, and genetic amniotic fluid analysis, which revealed a normal male karyotype (46,XY) for both fetuses.

Follow-up ultrasound seven days after the procedure demonstrated two viable fetuses with appropriate interval growth and normalization of Doppler findings. Fetus 1 showed an amniotic fluid DVP of 7.6 cm, normal UA and MCA Doppler indices, and a CPR of 1.022 (below the 5th percentile). Fetus 2 was in transverse lie with normal biometry, DVP of 5.0 cm, normal Doppler findings, and a CPR of 1.171. Both fetuses showed improvement in ductus venosus flow parameters, with positive a-wave and PIV values within the normal range (0.850 and 0.720, respectively).

The patient continued close weekly surveillance until delivery. At 36 weeks of gestation, a cesarean section was performed due to MCDA twin pregnancy post-laser therapy, post-cesarean section, with fetus 1 in breech presentation. Both neonates were delivered in good condition, weighing 2400 g and 2410 g, with Apgar scores of 8/10/10 and 9/10/10, respectively. The postoperative maternal course was uneventful, and the patient was discharged in good condition on the fourth postoperative day.

## Discussion

Early diagnosis and close surveillance are essential for improving perinatal outcomes in MCDA twin pregnancies, which are predisposed to vascular complications such as TTTS and TAPS. According to international guidelines, including the Society for Maternal-Fetal Medicine (SMFM) Consult Series #72, ultrasound monitoring is recommended every two weeks from 16 weeks of gestation onward to evaluate amniotic fluid, bladder filling, and Doppler studies of the UA, ductus venosus (DV), and MCA [8]. Such an approach allows early recognition of intertwin imbalances and timely referral to a specialized fetal therapy center before irreversible hemodynamic deterioration occurs.

The decision to perform fetoscopic laser photocoagulation is commonly guided by the Quintero staging system, which stratifies the severity of TTTS based on ultrasound and Doppler criteria (Table 1). Although this system has certain limitations, particularly its inability to fully predict outcomes following laser therapy, it remains the most widely applied clinical tool in defining the threshold for intervention [7,8].

**Table 1:** Quintero staging of twin-to-twin transfusion syndrome DVP: deepest vertical pocket; UA: umbilical artery; DV: ductus venosus; UV: umbilical vein

Quintero Stage	Diagnostic Criteria
Stage I	Oligohydramnios in the donor (DVP <2 cm) and polyhydramnios in the recipient (DVP >8 cm), with a visible donor bladder.
Stage II	Donor bladder not visible, Dopplers normal.
Stage III	Abnormal Doppler in either twin (UA, DV, or UV).
Stage IV	Hydrops fetalis in one or both fetuses.
Stage V	Intrauterine demise of one or both fetuses.

In the presented case, the pregnancy exhibited marked polyhydramnios in the recipient twin (DVP 13 cm), associated with maternal discomfort and early Doppler signs of increased resistance in the ductus venosus (PIV 1.430) with a preserved a-wave. The MCA-Vmax was reduced, and the CPR was 0.666 (<1.0), suggesting the beginning of hemodynamic compromise consistent with the onset of the brain-sparing effect, indicating early fetal adaptation to hypoxemia.

Conversely, the donor twin presented an amniotic fluid volume at the lower limit of normal (DVP 4 cm), with a visible bladder and positive a-wave, thus not fulfilling the strict criteria for Stage I TTTS. Additionally, the intertwin difference in MCA-PSV suggested borderline TAPS, but diagnostic thresholds were not fully met [6].

Based on the current SMFM and the International Society of Ultrasound in Obstetrics and Gynecology (ISUOG) recommendations, fetoscopic laser coagulation is primarily indicated for Stage II-IV TTTS between 16 and 26-28 weeks of gestation, while selective Stage I cases may also benefit from intervention when accompanied by significant maternal symptoms or early signs of hemodynamic imbalance [2]. In our case, the decision for early referral and laser therapy was influenced not only by the patient’s symptomatic polyhydramnios and Doppler alterations, but also by her high-risk maternal profile. The combination of a previous pregnancy complicated by severe preeclampsia with IUGR and pre-existing chronic hypertension under treatment increased the likelihood of maternal compromise under progressive polyhydramnios. Considering this background, the multidisciplinary team opted for an early fetoscopic laser procedure as the most balanced approach to prevent further maternal deterioration and to optimize perinatal outcomes.

Because fetoscopic laser therapy was not available at the Fetal-Maternal Medicine Department of the University Hospital of Vienna, the patient was transferred to the University Hospital of Graz, where laser ablation of placental anastomoses and amnioreduction were performed with rapid post-procedural improvement. Post-laser surveillance was conducted weekly during the first six weeks, a period associated with the highest risk for recurrent TTTS, post-laser TAPS, or growth discordance [8]. Stabilization of Doppler indices and amniotic fluid volumes enabled safe continuation of the pregnancy to 36 weeks, when cesarean delivery resulted in two healthy neonates.

The favorable maternal and neonatal outcome in this case emphasizes the value of individualized clinical judgment and multidisciplinary collaboration. Even when strict diagnostic thresholds for TTTS and TAPS are not met, early recognition of maternal-fetal compromise and timely referral to an experienced fetal surgery center can significantly improve survival and long-term outcomes.

## Conclusions

This case underscores the significance of early detection and multidisciplinary management in MCDA twin pregnancies complicated by borderline TTTS and TAPS. While classical criteria for fetoscopic laser therapy were not completely fulfilled, proactive clinical decision-making based on subtle hemodynamic changes and maternal symptoms led to successful perinatal outcomes. Continuous surveillance every two weeks, adherence to standardized Doppler assessment, and timely access to specialized fetal therapy centers remain the cornerstone of optimal care for monochorionic twin gestations.
